# Bidirectional associations between psychosocial well-being and adherence to healthy dietary guidelines in European children: prospective findings from the IDEFICS study

**DOI:** 10.1186/s12889-017-4920-5

**Published:** 2017-12-14

**Authors:** Louise Arvidsson, Gabriele Eiben, Monica Hunsberger, Ilse De Bourdeaudhuij, Denes Molnar, Hannah Jilani, Barbara Thumann, Toomas Veidebaum, Paola Russo, Michael Tornatitis, Alba M. Santaliestra-Pasías, Valeria Pala, Lauren Lissner, IDEFICS consortium

**Affiliations:** 1 Section for Epidemiology and Social Medicine (EPSO), The Sahlgrenska Academy, University of Gothenburg, Box 453, 405 30 Gothenburg, Sweden; 2 Department of Movement and Sport Sciences, Ghent University, Ghent, Belgium; 3 Department of Paediatrics, Clinical Center, University of Pécs, Pecs, Hungary; 4 Leibniz Institute for Prevention Research and Epidemiology – BIPS, Achterstrasse 30, 283 59 Bremen, Germany; 5 National Institute for Health Development, Tallinn, Estonia; 6 Institute of Food Sciences, CNR Via Roma, 64-83100 Avellino, Italy; 7 Research and Education Institute of Child Health REF, Strovolos, Cyprus; 8 GENUD (Growth, Exercise, Nutrition, and Development) Research group, University of Zaragoza; Instituto Agroalimentario de Aragón (IA2); Instituto de Investigación Sanitaria Aragón (IIS Aragón); Centro de Investigación Biomédica en Red de Fisiopatología de la Obesidad y Nutrición (CIBERObn), Zaragoza, Spain; 9 Nutritional Epidemiology Unit, Department of Preventive Medicine, Fondazione IRCSS Istituto Nazionale dei Tumori, Milan, Italy

**Keywords:** IDEFICS, Dietary guidelines, Healthy diet score, Psychosocial well-being, Childhood overweight

## Abstract

**Background:**

In children the relationship between a healthy diet and psychosocial well-being has not been fully explored and the existing evidence is inconsistent. This study investigates the chronology of the association between children’s adherence to healthy dietary guidelines and their well-being, with special attention to the influence of weight status on the association.

**Methods:**

Seven thousand six hundred seventy five children 2 to 9 years old from the eight-country cohort study IDEFICS were investigated. They were first examined between September 2007 and June 2008 and re-examined again 2 years later. Psychosocial well-being was measured using self-esteem and parent relations questions from the KINDL® and emotional and peer problems from the Strengths and Difficulties Questionnaire. A Healthy Dietary Adherence Score (HDAS) was calculated from a 43-item food frequency questionnaire as a measure of the degree to which children’s dietary intake follows nutrition guidelines. The analysis employed multilevel logistic regression (country as random effect) with bidirectional modeling of dichotomous dietary and well-being variables as both exposures and outcomes while controlling for respective baseline values.

**Results:**

A higher HDAS at baseline was associated with better self-esteem (OR 1.2, 95% CI 1.0;1.4) and fewer emotional and peer problems (OR 1.2, 95% CI 1.1;1.3 and OR 1.3, 95% CI 1.2;1.4) 2 years later. For the reversed direction, better self-esteem was associated with higher HDAS 2 years later (OR 1.1 95% CI 1.0;1.29). The analysis stratified by weight status revealed that the associations between higher HDAS at baseline and better well-being at follow-up were similar in both normal weight and overweight children.

**Conclusion:**

Present findings suggest a bidirectional relation between diet quality and self-esteem. Additionally, higher adherence to healthy dietary guidelines at baseline was associated with fewer emotional and peer problems at follow-up, independent of children’s weight status.

**Electronic supplementary material:**

The online version of this article (10.1186/s12889-017-4920-5) contains supplementary material, which is available to authorized users.

## Background

In 2005 the Mental Health Foundation recognized diet as an underestimated determinant of mental health [1]. Studies have found both suppression of negative emotions and inability to withstand negative emotions to be associated with excessive food intake, particularly of foods rich in fat and sugar, often considered comfort foods [2, 3]. Furthermore, emotional state may influence taste: stress or negative emotions have been reported to diminish sweet taste and enhance sour taste while the opposite was found for positive emotions [4]. Macht *el al* have suggested a bidirectional relationship where emotions regulate eating, and eating regulates emotions [5]. This theory was further advanced by Singh et al. who suggested a cycle of negative emotions leading to excess intake of comfort foods which in time leads to obesity that furthers the negative emotional state due to metabolic disturbances [6].

In children the evidence for an association between diet and psychosocial well-being is limited and inconsistent. In one study, high intake of sugar-rich foods was associated with higher odds of emotional problems, while higher diet quality (low in fat but high in plant foods) was associated with lower odds of emotional problems [7]. Similarly, energy-dense and nutrient-poor diets were cross-sectionally associated with hyperactivity-inattention disorders and conduct/opposition disorders, while a diverse diet rich in plant foods and fish was associated with lower odds of having psychiatric and hyperactivity-inattention disorders [8]. Recently a review by O’Neil et al. found consistent evidence of a cross-sectional association between unhealthy diets (generally including refined grains, processed meat and snacks, diet- and sugar rich soft drinks, fried food and foods high in saturated fat and sugar) and poor psychosocial well-being in children and adolescents [9]*.* Little is known about the longitudinal relation between diet and behavioral problems in children, although one study [10] found no associations between high intakes of processed snack foods and other foods high in fat and/or sugar in relation to behavioural problems 16 months later. However, one has to acknowledge the possible effect of children’s weight status on the association between diet and psychosocial well-being [6, 11]. For example, recent results from the IDEFICS (Identification and Prevention of Dietary- and Lifestyle-Induced Health Effects in Children and Infants study) suggests that childhood overweight increases the risk of poor health-related quality of life while poor well-being increases the risk of developing overweight [12].

There is a lack of prospective research investigating the link between food intake and psychosocial well-being in children. More specifically, the association between healthy dietary intake and children’s well-being needs to be confirmed and investigated for chronology and potential causality. Hence, the primary aim of this study is to investigate the bidirectional association between adherence to healthy dietary guidelines and children’s psychosocial well-being and the extent to which these associations might differ between children with different weight status.

## Methods

### Participants

The present study includes children from the Identification and Prevention of Dietary- and Lifestyle-Induced Health Effects in Children and Infants study (IDEFICS). IDEFICS is a prospective cohort study with an embedded intervention, including eight European countries (Belgium, Cyprus, Estonia, Germany, Hungary, Italy, Spain and Sweden). The general purpose of IDEFICS is to understand how to prevent overweight in children while considering its multifactorial etiology. Ethics approval was obtained from review boards responsible in each country. Parents provided written informed consent, and children gave oral consent for examinations and sample collection. Further information about the IDEFICS study can be obtained from previous publications [13, 14].

**Fig. 1:**
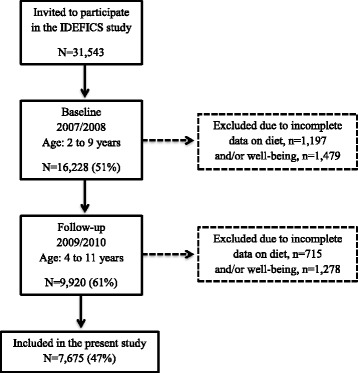
Flowchart on participants included in the present study

Originally, 16,228 children aged 2 to 9 years participated at IDEFICS baseline (September 2007 to June 2008). The baseline survey was followed by a community intervention in half of the sample and then a 2 years follow-up examination was conducted in 9920 children from the original baseline cohort. The prospective design required that the same instruments and examinations were employed at both time points. To assure quality and comparability across research centers a translation/back-translation for each local language was performed, together with a re-administration to a sub-sample for assessing reliability [13]. Data on diet and indicators of well-being were obtained by parental proxy reporting using questionnaires. Only children with complete data on diet and indicators of well-being from both time points were included, hence the final sample consisted of 7675 children, 51% males (Fig. 1).

### The healthy dietary adherence score (HDAS)

An a priori diet score, the Healthy Dietary Adherence Score (HDAS), was calculated from a 43-item food frequency questionnaire (FFQ). At baseline and follow-up parents (or other caregivers) were asked to report the usual consumption frequency in a typical week during the preceding 4 weeks for all meals consumed at home or in the presence of the parents excluding e.g. foods served at school: *In the last month, how many times did your child eat or drink the following food items?* Possible options for answering were: *never/less than once a week, 1–3 times a week, 4–6 times a week, 1 time per day, 2 times per day, 3 times per day, 4 or more times per day, I have no idea*. Children for whom more than 21 (50%) of the FFQ items were missing were excluded. Based on this definition the rate of complete FFQ (less than 50% of food items missing) were 93% at both baseline and follow-up. In the remaining sample of children the missing food items as well as the answer ‘I have no idea’ were treated as not consumed when creating composite scores. This is a common practice in nutrition surveys because food items are often left blank if not consumed [15–17]. A pilot study found the FFQ to be reproducible with mean Kappa coefficients ranging from 0.41 to 0.60 and Spearman’s correlation higher than 0.5 for 81% of the food items [18]. Further, a validation study against repeated 24-h dietary recall found that under 12% of the food groups were classified in the wrong quartile of intake [19].

The HDAS was developed to reflect the guidelines established by Waijers et al. [20, 21]. Specifically, the HDAS aimed to capture adherence to healthy dietary guidelines common for all eight countries participating in the IDEFICS study. Moreover, the design of the HDAS allows for a standardization of number of foods and beverages reported, and consumption frequency, in order to avoid misclassification of children into low or high adherence just because they consume all types of food frequently. The guidelines included: limit the intake of refined sugars, reduce fat intake, especially of saturated fat, choose whole meal when possible, consume 400–500 g of fruits & vegetables per day and fish 2–3 times per week. Hence, the HDAS contains five components: sugar, fat, whole meal, fruits & vegetables, and fish. Each component has a minimum score of 0 and a maximum score of 10, summed to a maximum score of 50, where the highest score indicates the highest possible adherence to the dietary guidelines. Both the HDAS and its components were dichotomized into “lower adherence” and “higher adherence” at the group median with the median included in the higher adherence group. A more detailed description of the HDAS can be found in Additional file 1.

### Indicators of psychosocial well-being

Four indicators of psychosocial well-being (referred to as well-being) were examined at baseline and follow-up, namely self-esteem, parent relations, emotional and peer problems. Self-esteem and parent relations were calculated from responses to the validated Kinder Lebensqualität Fragebogen (KINDL®) [22–24]. The IDEFICS study included a version of the KINDL® developed for parent response on behalf of children and adolescents between 7 and 17 years of age. The self-esteem score included: *During the last week my child…* (1) *had fun and laughed a lot*, (2) *didn’t feel much like doing anything,* (3) *felt alone,* and (4) *felt scared or unsure of him/herself*. The parent relations score included: *During the last week my child…* (1) *got on well with us as parents*, (2) *felt fine at home,* (3) *we quarreled at home,* and (4) *felt that I was bossing him/her around*. The items were scored from 1 (never) to 4 (often or always) with reversals according to the wording of the question, summed to total scores and transformed to percentage scores ranging from 0 to 100%. The total scores where then dichotomized into ‘poor’ or ‘good’ using sex- and age-specific cut-off scores from the KINDL® manual [22]. However, self-esteem was later re-categorized into ‘lower’ and ‘better’ scores at the group median since a majority of the children (98% at baseline and 97% at follow-up) reported ‘good’ scores suggesting that our population experienced higher self-esteem compared to the reference population of corresponding sex- and age-groups.

Emotional and peer problems were calculated from Goodmann et al.’s validated Strengths and Difficulties Questionnaire (SDQ) [25–27] developed for children ages 4 to 16 years. The IDEFICS study used the informant-rated version which has been found to correlate well with the child-rated version [27, 28]. The peer problem score included: *To what extent do the following characterizations apply to your child?* (1) *rather solitary, tends to play alone, (2) has at least one good friend, (3) generally liked by other children (4) picked on or bullied by other children, and (5) gets on better with adults than with other children.* The emotional problem score included: *To what extent do the following characterizations apply to your child?* (1) *often complains of headaches, stomach-aches or sickness,* (2) *many worries or often seems worried,* (3) o*ften unhappy, depressed or tearful,* (4) *nervous in new situations, easily loses confidence,* and (5) *many fears, easily scared.* Items were scored from 0 ‘not true’ to 2 ‘certainly true’ and summed to total scores ranging from 0 to 10 where a high value indicated more difficulties or life struggles. In accordance with the SDQ manual [28] the emotional and peer problem scores were divided into: ‘inconspicuous’, ‘borderline’ and ‘abnormal’. Thereafter, a dichotomized variable was created, as previously done by Hunsberger et al. [12], consisting of poor well-being (including both ‘borderline’ and ‘abnormal’ groups) versus the remaining children with no detectable (‘inconspicuous’) poor wellbeing.

### Covariates

Measured anthropometrics were collected at both baseline and follow-up. Weight was measured to the nearest 0.1 kg with a Tanita BC 420 SMA scale and height was measured to the nearest 0.1 cm by a SECA 225 Stadiometer. Examinations were conducted in the morning, with the children fasting and in light clothing. Body Mass Index (BMI) and age-and sex specific BMI z-scores and cut-points for children and adolescents developed by the International Obesity Task Force (IOTF) [29] were calculated and used to categorize children as normal weight (including thin) or overweight (including obese).

Data on parental education and income was collected from the parental questionnaire. The education level is based on the International Standard Classification of Education (ISCED) for cross-country comparability and was used to determine the highest level of either parents’ education [30]. Levels 1–3 represent upper secondary education (classified as lower education level) and levels 4–6 represent post-secondary education (classified as higher education level). Country-specific income levels were assigned with reference to the average net equivalence income, considering the median income and poverty line. Levels 1–5 represent lower income level and levels 6–9 represent higher income level.

### Statistics

Descriptive characteristics are presented as mean, standard deviation, minimum and maximum for continuous variables (age, BMI z-score, indicators of well-being and the HDAS scores), and number and percentage for categorical variables (sex of the child, weight status, SEP, and categories of well-being, HDAS and its components). Due to the hierarchical structure of the data Generalized Linear Mixed Models (GENLINMIXED) were used to analyze the prospective association between higher adherence to the HDAS and its components at baseline and indicators of well-being 2 years later. Random intercepts for country were included to consider the clustered study design. The model was adjusted for age, sex, BMI z-score, baseline well-being, and highest parental education and income. To investigate directionality, associations between well-being at baseline and adherence to the HDAS and its components 2 years later were analyzed using the same procedure, now adjusting for baseline diet factors.

A sensitivity analysis was performed to further explore the chronology of associations between the HDAS and well-being. In these analyses, group medians were used as cut-off for the indicators of well-being (as both exposure and outcome) to estimate standardized effect sizes in both directions. The relationship with parents was dichotomized into ‘lower’ and ‘higher’ scores (median included in the higher group), with a higher score indicating higher well-being. Furthermore, emotional and peer problems were dichotomized into ‘lower’ and ‘higher’ scores (median included in the lower group), and here a lower score indicated higher well-being. As previously described, self-esteem was already dichotomized based on group median. An additional sensitivity analysis using quartiles of adherence to the HDAS was performed in order to further explore the potential dose-response relationship between baseline diet and children’s subsequent well-being.

Next, stratified analyses were performed to investigate if the association between diet and well-being differed between children with overweight compared to children with normal weight. Finally, a drop-out analysis was conducted to compare children included in the present study with those who only participated in the IDEFICS baseline measurements. Student t-test was used for continuous variables (age, BMI z-score, the HDAS) and Pearson’s χ^2^-test to compare categorical variables (sex of the child, parental education, parental income, weight status, indicators of well-being). All analyses were performed with IBM SPSS Statistics Version 20. The significance level was set to 0.05.

## Results

As presented in Table 1, the analytic sample contained a similar number of boys and girls. At baseline, the mean age was 6.0 (±1.8) and the mean BMI z-score was 0.3 (±0.3). On a group level, the Healthy Dietary Adherence Score (HDAS) and all indicators of well-being were stable between baseline and follow-up. However, the prevalence of overweight (including obesity) in these children increased from 18% to 23% during the two-year follow up, according to the criteria of Cole et al. [29]. Finally, at baseline 59% of the children had parents’ with post-secondary education and 52% of the children had parents with an income above the country-specific median (based on centrally calculated national statistics from each country).

**Table 1:** Descriptive characteristics at baseline and follow-up in the complete sample (*N* = 7675)

	Baseline 2007/2008			Follow-up 2009/2010		
	*mean ± SD*	*min., max.*	*IQR*	*mean ± SD*	*min., max.*	*IQR*
Age (years)	6.0 (1.8)	2.0;9.7	4.4;7.6	8.0 (1.8)	3.9;11.8	6.4;9.5
BMI z-score^1^	0.3 (1.2)	−5.4;5.8	−0.5;1.0	0.4 (1.2)	−6.6;4.7	−0.4;1.2
Self-esteem score (KINDL®)	89.4 (10.4)	25.0;100.0	81.3;100.0	87.0 (10.7)	25.0;100.0	81.3;93.8
Parent relations score (KINDL®)	84.8 (10.2)	43.8;100.0	75.0;93.8	83.9 (10.5)	25.0;100.0	75.0;93.8
Emotional problems score (SDQ)	1.6 (1.7)	0.0;10.0	0.0;2.0	1.6 (1.7)	0.0;10.0	0.0;2.0
Peer problems score (SDQ)	1.3 (1.5)	0.0;10.0	0.0;2.0	1.2 (1.5)	0.0;10.0	0.0;2.0
HDAS	22.0 (9.0)	0.0;49.0	16.0;28.0	23.0 (9.0)	0.0;49.0	17.0;28.0
	*n*	*%*		*n*	*%*	
Sex of the child (male)	3894	51		3894	51	
Overweight (including obese)	1385	18		1727	23	
Higher parental education level^2^	4480	59		4572	60	
Higher parental income level^3^	3738	52		4259	60	
*Indicators of well-being*						
Better self-esteem (KINDL®)^Δ^	4086	53		4702	61	
Good parent relations (KINDL®)^Δ^	3914	51		3824	50	
No detectable emotional problems (SDQ)^Λ^	6625	86		6620	86	
No detectable peer problems (SDQ)^Λ^	6194	81		6349	83	
*Healthy Dietary Adherence Score*						
Total HDAS (higher adherence)†	4017	52		3970	52	
*HDAS components (higher adherence)*						
fruits & vegetables	4983	65		4993	65	
fish	5383	71		5315	70	
whole meal	4011	52		4217	56	
sugar	1642	21		1868	24	
fat	4846	63		4196	55	

^1^BMI z-score calculated based on the method proposed by the International Obesity Task Force [29]

^2^Post-secondary education

^3^Country-specific reference to the average net equivalence income, considering the median income and poverty line

^Δ^Cut-off values for Better self-esteem ≥ 87.50 (both sexes) and Good parent relations > 83.58(m)/84.40(f)

^Λ^Cut-off values for No detectable emotional problems ≤ 3 and No detectable peer problems ≤ 2

^†^Cut-off value for Higher adherence to the HDAS ≥ 21 at baseline and ≥ 22 at follow-up

*IQR* Interquartile Range; *SD* standard deviation; *SDQ* Strengths and Difficulties Questionnaire; *HDAS* Healthy Dietary Adherence Score

### Associations between baseline diet and well-being at follow-up

Table 2 presents the results from the multilevel analysis with higher versus lower well-being as the outcome. Higher HDAS at baseline was associated with higher self-esteem (OR 1.2, 95% CI 1.0;1.4) and fewer emotional or peer problems (OR 1.2, 95% CI 1.1;1.3 and OR 1.3, 95% CI 1.2;1.4) 2 years later. Further analyses of the components included in the HDAS identified positive associations between baseline consumption of fruits & vegetables, fish, whole meal and fat in accordance with dietary guidelines and indicators of well-being 2 years later.

**Table 2:** Prospective associations between the Healthy Dietary Adherence Score at baseline and indicators of psychosocial wellbeing at follow-up (*N* = 7196)

	KINDL®				SDQ			
	Better self-esteem		Good parent relations		No detectable emotional problems		No detectable peer problems	
	OR	95% CI	OR	95% CI	OR	95% CI	OR	95% CI
HDAS (higher adherence)	1.2**	1.1–1.3	1.1	1.0–1.3	1.2**	1.1–1.3	1.3***	1.1–1.4
*HDAS components (higher adherence)*								
Fruit & Vegetables	1.2*	1.0–1.3	1.3**	1.1–1.4	1.2*	1.0–1.3	1.2**	1.0–1.3
Fish	1.2*	1.0–1.4	1.1	1.0–1.3	1.2**	1.1–1.4	1.2**	1.1–1.4
Whole meal	1.1	0.9–1.3	1.0	0.9–1.1	1.1	0.9–1.2	1.1*	1.0–1.3
Sugar	1.1	0.9–1.3	1.2	1.0–1.4	0.9	0.8–1.1	1.0	0.9–1.2
Fat	1.0	0.9–1.2	1.0	0.9–1.2	1.2**	1.1–1.3	1.1	1.0–1.2

*p < 0.05, **p < 0.01, ***p < 0.001

*P*-value obtained using a multilevel model correcting for cluster design (country)

Model adjusted for: age, sex, BMI z-score, well-being at baseline, and highest parental education and income

*HDAS* Healthy Dietary Adherence Score; *OR* Odds ratio; *SDQ* Strengths and Difficulties Questionnaire

### Associations between baseline well-being and diet at follow-up

The analysis was then repeated to study adherence to healthy dietary guidelines as the outcomes, measured by the HDAS and its components (Table 3). The fully adjusted model identified better self-esteem to be associated with higher HDAS 2 years later (OR 1.1, 95% CI 1.0;1.3). Additional analysis of the components included in the HDAS found positive associations between baseline indicators of well-being and consumption of fruits & vegetables, sugar and fat in accordance with dietary guidelines 2 years later.

**Table 3:** Prospective associations between indicators of well-being at baseline and the Healthy Dietary Adherence Score at follow-up (*N* = 7196)

				*HDAS components (higher adherence)*									
		HDAS (higher adherence)		Fruits & Vegetables		Fish		Whole meal		Sugar		Fat	
		OR	95% CI	OR	95% CI	OR	95% CI	OR	95% CI	OR	95% CI	OR	95% CI
KINDL®	Better self-esteem	1.1*	1.0–1.3	1.1	1.0–1.2	1.0	0.9–1.2	1.1	1.0–1.3	1.2**	1.1–1.4	1.0	0.9–1.1
	Good parent relations	1.0	0.9–1.1	1.2*	1.0–1.3	1.0	0.9–1.1	1.0	0.9–1.2	1.0	0.9–1.2	1.1	0.9–1.2
SDQ	No detectable emotional problems	1.1	1.0–1.3	1.1	0.9–1.2	1.1	1.0–1.3	1.0	0.9–1.2	1.1	0.9–1.3	1.2*	1.0–1.4
	No detectable peer problems	1.0	0.9–1.1	1.2**	1.1–1.4	1.1	0.9–1.2	1.1	1.0–1.3	1.1	0.9–1.3	1.0	0.9–1.2

*p < 0.05, **p < 0.01

*P*-value obtained using a multilevel model correcting for cluster design (country)

Model adjusted for: age, sex, BMI z-score, diet at baseline, and highest parental education and income

*HDAS* Healthy Dietary Adherence Score; *OR* Odds ratio; *SDQ* Strengths and Difficulties Questionnaire

Results from the sensitivity analysis using group median as cut-off for the indicators of well-being did not identify any further bidirectional associations (results not shown). Since self-esteem was already dichotomized based on group median the bidirectional association previously established between self-esteem and HDAS remained unchanged.

### Sensitivity analysis

The dichotomization of the Healthy Dietary Adherence score into “lower adherence” and “higher adherence” at the group median was a subjective decision since no clinical cut-off exists for what would define an adequate adherence to these guidelines. However, to further explore the association between baseline diet and children’s well-being at follow-up, a sensitivity analysis was performed using quartiles of adherence to the HDAS. Although no clear dose-response relation could be identified there was a monotonic trend in the odds of having better well-being for higher adherence to the HDAS. In comparison to children in the lowest quartile (i.e. lowest adherence to the HDAS), children in the highest quartile had 1.2 times higher odds of reporting better self-esteem, 1.4 times higher odds of reporting less emotional problems and 1.3 times higher odds of reporting good peer relations.

### Prospective associations across weight groups

As presented in Table 4, higher adherence to the HDAS at baseline was associated with fewer emotional and peer problems (OR 1.2, 95% CI 1.1;1.4 and 1.3, 95% CI 1.1;1.4) in the group of children with normal weight (*n* = 5948). Further, among the group of children with overweight (*n* = 1727) a higher adherence to the HDAS was associated with fewer peer problems (OR 1.4, 95% CI 1.1–1.7). For the reversed direction, i.e. baseline well-being and diet at follow-up, no significant associations were found either in the normal weight or overweight group (Tables 5 and 6).

**Table 4:** Sensitivity analysis of the prospective associations between adherence to the Healthy Dietary Adherence Score (divided into quartiles) at baseline and indicators of psychosocial well-being at follow-up (*N* = 7196)

	KINDL®				SDQ			
	Better self-esteem		Good parent relations		No detectable emotional problems		No detectable peer problems	
HDAS^†^	OR	95% CI	OR	95% CI	OR	95% CI	OR	95% CI
Q4 (>28)	1.2	1.0–1.5	1.2	1.0–1.5	1.4	1.2–1.6	1.3	1.1–1.6
Q3 (21–27)	1.2	1.0–1.4	1.1	0.9–1.3	1.3	1.1–1.5	1.3	1.1–1.4
Q2 (16–20)	0.9	0.8–1.1	1.1	0.9–1.3	1.2	1.1–1.4	1.0	0.9–1.2
Q1 (<15)	reference		reference		reference		reference	

OR and CI obtained using a multilevel model correcting for cluster design (country)

Model adjusted for: age, sex, BMI z-score, well-being at baseline, and highest parental education and income

^†^HDAS ranges from 0 to 49

*HDAS* Healthy Dietary Adherence Score; *OR* Odds ratio; *SDQ* Strengths and Difficulties Questionnaire

**Table 5:** Prospective association between the Healthy Dietary Adherence Score at baseline and indicators of well-being at follow-up across weight groups at follow-up

			KINDL®				SDQ			
			Better self-esteem		Good parent relations		No detectable emotional problems		No detectable peer problems	
			OR	95% CI	OR	95% CI	OR	95% CI	OR	95% CI
HDAS (higher adherence)	Weight group	I	1.2	1.0–1.4	1.1	1.0–1.3	1.2**	1.1–1.4	1.3***	1.1–1.4
		II	1.2	0.9–1.7	1.2	0.9–1.5	1.2	1.0–1.6	1.4**	1.1–1.7

**p < 0.01, ***p < 0.001

*P*-value obtained using a multilevel model correcting for cluster design (country)

Weight group: I = normal weight including thin, *n* = 5597, II = overweight including obese, *n* = 1599

Model adjusted for: age, sex, BMI z-score, well-being at baseline, and highest parental education and income

*HDAS* Healthy Dietary Adherence Score; *OR* Odds ratio; *SDQ* Strengths and Difficulties Questionnaire

**Table 6:** Prospective association between indicators of well-being at baseline and the Healthy Dietary Adherence Score at follow-up across weight groups

		HDAS (higher adherence)		
		Weight group	OR	95% CI
KINDL®	Better self-esteem	I	1.1	1.0–1.3
		II	1.2	0.9–1.4
	Good parent relations	I	1.0	0.9–1.2
		II	1.1	0.9–1.4
SDQ	No detectable emotional problems	I	1.2	1.0–1.4
		II	1.2	0.9–1.6
	No detectable peer problems	I	0.9	0.8–1.0
		II	1.2	0.9–1.6

*P*-value obtained using a multilevel model correcting for cluster design (country)

Weight group: *I* = normal weight including thin, *n* = 5597, *II* = overweight including obese, *n* = 1599

Model adjusted for: age, sex, BMI z-score, diet at baseline, and highest parental education and income

*HDAS* Healthy Dietary Adherence Score; *OR* Odds ratio; *SDQ* Strengths and Difficulties Questionnaire

### Drop-out analysis

A drop-out analysis was conducted to compare children who remained in the study (*N* = 7675) and children who were lost to follow-up (*N* = 8553). As previously reported from the IDEFICS study [31] children who attended follow-up examinations had a lower prevalence of overweight (18 vs. 21% *p* < 0.001) and were more likely to be from families with higher parental education (61 vs. 58% *p* < 0.001) and have parents with higher income (52 vs. 38%). Additionally, they were more likely to have better self-esteem (98 vs. 86% *p* < 0.001) and good parent relations (51 vs. 42%), and had fewer emotional (86 vs. 85% *p* < 0.01) and peer problems (81 vs. 76% *p* < 0.001). Compared to the children who dropped out, the continued participants had a somewhat higher total HDAS (22 (8.9) vs. 21.3 (8.9) *p* < 0.001). There was no difference in age or sex of the children between the two groups.

## Discussion

In this study we documented a bidirectional association between adherence to healthy dietary guidelines and children’s self-esteem. Furthermore, prospective associations were found between higher Healthy Dietary Adherence Score (HDAS) at baseline and less emotional and peer problems at follow-up. These associations could not be explained by children’s weight status. These findings are unique in that they are based on a large longitudinal study of children from different parts of Europe, adding to the largely cross-sectional, current evidence on diet and psychosocial health in children [9].

### Associations between baseline diet and well-being at follow-up

The associations between higher adherence to the HDAS and better well-being in children support previously reported connections between diet and mental health in adolescent populations [8, 32, 33]. Prospective associations between diet quality and mental health were reported by Jacka et al. [32]. They found that increased diet quality was associated with improved mental health during a 3-year follow-up, while a decrease in diet quality was followed by a decline in mental health (measured using the emotional subscale of the Pediatric Quality of Life Inventory) [32]. In contrast to our findings, no bidirectional association was identified by Jacka et al., i.e. no significant association was found between mental health status at baseline and diet quality 3 years later [32]. Further, Kulkarni et al. reported independent effects of healthy and unhealthy diets on adolescent mental health, which is important because, as stated by the author, “an increase in one of these behaviors is not necessarily indicative of a decrease in the other” [33]. Some evidence exists that dietary interventions improve symptoms of depression [34]. However, a recent study by Kaseva et al. reported no difference in psychological well-being at the age of 20 in participants (as compared to controls) after a repeated dietary and lifestyle intervention [35].

This study is unique in that associations between components of the HDAS and well-being were explored. Fruit & vegetable consumption according to guidelines (400–500 g per day) was associated with all indicators of good well-being; fish intake according to the guidelines (2–3 times per week) was associated with better self-esteem and no emotional or peer problems; and finally, intake of whole meal according to guidelines was associated with no peer problems. These findings reflect the dietary recommendations for the prevention of depression in adults that were recently published by Opie et al. [36] and suggest that these foods could be important for psychosocial well-being in younger populations. Since the indicators of well-being are measures of coping responses to external stressors like e.g. demand, challenges or events [37] the present findings could indicate that a healthy diet is an important factor for coping ability in children.

These findings are fully consistent with current knowledge and assumptions that children’s physical and mental development is dependent on nutritional quality [38]. Moreover, by categorizing children into quartiles of adherence to the HDAS we identified a monotonic trend in the odds of having better well-being. Although it is beyond the scope of the present study to draw any conclusions about the mechanism(s) that links diet and well-being one could hypothesize the biological significance of omega-3 fatty acids and the micronutrient content of the diet may positively impact mental health [39, 40]. Additionally, one should consider the importance of a healthy diet pattern on other lifestyle factors such as e.g. dental health [41], and sleep [42, 43] and that the association between diet and well-being might reflect an overall good health in these children.

### Associations between baseline well-being and diet at follow-up

Among adult populations studies suggest that depression as well as negative emotions and inability to withstand negative emotions are associated with consumption of foods rich in fat and sugar [2, 3, 44]. Furthermore, depression has been associated with lower consumption of fruits and vegetables [45]. To date, the prospective effect of psychosocial well-being on dietary intake in children has hardly been investigated. Previous cross-sectional studies have related poor mental health to poor eating habits [9] such as high intake of fast food, fried food and lower intake of vegetables [46]. Additionally, a previous study by Michels et al. identified a cross-sectional association between behavioral problems in children and frequent consumption of foods rich in fat and sugar [47]. The present study adds to the existing literature in that we identified a prospective association between better self-esteem at baseline and a healthier diet at follow-up. Furthermore, associations were established between baseline indicators of well-being and components included in the HDAS; better self-esteem at baseline was associated with sugar intake in accordance with the guidelines (limited intake of refined sugars); good parent relations was associated with fruit & vegetable consumption according to guidelines (400–500 g per day); fewer emotional problems was associated with fat intake according to the guidelines (reduced intake, especially of saturated fat); and finally fewer peer problems was associated with consumption of fruits & vegetables according to guidelines.Previous findings of poor well-being relating to unhealthy eating [9, 46, 47] could be due to the fact that emotional eating is a coping mechanism [48] and that the previously reported change in taste (where negative emotions diminish sweet taste while enhancing sour taste) [4] might affect food choices. In the present study the focus was on healthy eating habits and the positive associations found between baseline well-being and adherence to a healthy diet at follow-up could reflect the fact that children with better well-being have less need of food as a coping mechanism. However, it is important to consider the chronology of the associations identified. Bidirectional associations were found between children’s self-esteem and the HDAS. However, the effect estimates were similar for both directions: a higher HDAS at baseline was associated with 1.2 times higher odds ratio of better self-esteem, and conversely, better self-esteem at baseline was associated with 1.1 higher odds of a higher HDAS. This could indicate a possible positive reinforcement of a healthy diet on higher self-esteem and vice versa. Although these estimates were not significantly different, a general observation in the present study was more frequent associations between baseline diet and psychosocial well-being at follow-up rather than the reverse.

### The effect of weight status on the association between diet and well-being

Children with overweight and obesity constitute a vulnerable group when it comes to psychosocial well-being due to the frequent stigmatization because of their excess weight [49, 50]. These children are to a higher extent subject to bullying as compared to children with normal weight [51]. The Longitudinal Study of Australian Children (LSAC) identified poor psychosocial health (specifically regarding social and emotional functioning) among children with overweight [52]. Similarly, recent results from the IDEFICS study indicates that children with overweight are at higher risk of developing poor health related quality of life [12]. Therefore, the present finding of an association between higher adherence to the HDAS at baseline and better peer relations 2 years later independent of children’s weight status is an encouraging finding.

### Strengths and limitations

Our study adds to the existing literature in that we were able to examine the association between adherence to healthy dietary guidelines and indicators of well-being in a large European study of children during a two-year follow-up using the same measures. However, this study is not without limitations. In line with other prospective studies our drop-out analysis identified a selective non-participation at follow-up. The fact that children with poor diet and poor well-being were under-represented makes conclusion about prevalence or incidence problematic. Nevertheless, this type of participation bias does not preclude us from investigating the relationship between diet and well-being in this sample. Another possible limitation is that the SDQ and the KINDL® were designed for children from the age of 4 and 7 respectively, which is slightly older than some of the youngest children in our study. Further, parental reports of children’s diet and well-being were used due to the young age group; however, both the KINDL® and the SDQ have shown good inter-rater correlations between children and parent reports [23, 27]. Regarding the diet indices, it should be noted that the HDAS was based on healthy dietary guidelines common for the eight European countries included in the IDEFICS study, hence substantial evidence of good health effects are underlying the foods and beverages included. Additionally, the FFQ that is the basis of the HDAS has been validated and found to be reproducible [18, 19], although usual diet may still be biased with respect to social desirability. Finally, weight could act as both a confounder and mediator in the association between diet and well-being; hence all analyses were controlled for BMI, although there could still be residual confounding from e.g. fat mass and fat distribution not captured by BMI. Moreover, no data was available on family psychosocial disadvantage like e.g. parental well-being. Hence, there is still the possibility that the observed associations could be explained by residual confounding by aspects of both diet and well-being that our instruments were not able to capture. To end with the authors acknowledge that the high prevalence of the outcome better self-esteem close to 50% could lead to an overestimation of the odds ratios reported. However, this applies to both diet and self-esteem as outcome variable and should not affect the bi-directional comparability. Moreover, a sensitivity analysis using quartiles of adherence to the Healthy Dietary Adherence score could confirm a monotonic trend in all indicators of well-being with higher diet quality.

## Conclusion

We have shown evidence of prospective associations between higher adherence to healthy dietary guidelines and better well-being in a large European cohort study. In contrast to previous research we focused on a large number of healthy components of the children’s diet hence not exclusively unhealthy food consumption. Furthermore, a bidirectional association was identified between higher adherence to the Healthy Diet Adherence score and better self-esteem. These results are not limited to normal weight or overweight children and suggest a particularly positive role of a healthy diet on children’s well-being that should be considered in future research on psychosocial well-being in children.

## Additional file

Additional file 1: The Healthy Dietary Adherence Score (HDAS). (DOCX 24 kb)

## Abbreviations

BMI: Body Mass Index
CEHQ-FFQ: Children’s Eating Habits Questionnaire
DALY: Disability Adjusted Life Years
HDAS: Healthy Dietary Adherence Score
IDEFICS: Identification and Prevention of Dietary- and Lifestyle-Induced Health Effects in Children and Infants
IOTF: International Obesity Task Force
ISCED: International Standard Classification of Education
KINDL: Kinder Lebensqualität Fragebogen questionnaire
SDQ: Strengths and Difficulties Questionnaire
