# Supplementary material for: Bidirectional associations between psychosocial well-being and adherence to healthy dietary guidelines in European children: prospective findings from the IDEFICS study
Source: BMC Public Health. 2017 Dec 14;17:926. doi: 10.1186/s12889-017-4920-5 (PMC5729410; doi:10.1186/s12889-017-4920-5)
Supplement: Additional file 1: — The Healthy Dietary Adherence Score (HDAS). (DOCX 24 kb) [file 12889_2017_4920_MOESM1_ESM.docx]

**Additional file 1. The Healthy Dietary Adherence Score (HDAS)**

The HDAS was developed using total weekly frequency of specific foods and/or beverages divided by the child’s total reported consumption frequency of all foods and/or beverages. This provides standardization on number of foods and beverages reported and consumption frequency, avoiding misclassification of children into low or high adherence because they consume all types of food frequently. Moreover, this corrects for variation in eating occasions away from home that were not captured in the FFQ. Based on answers to the question “How often does your child usually eat at home or at other people’s home?” reports of less than 3 meals per week were excluded. The HDAS aimed to capture adherence to healthy dietary guidelines including: limiting the intake of refined sugars, reducing fat intake, especially of saturated fat, choosing whole meal when possible, consuming 400-500 gram of fruits & vegetables per day and fish 2-3 times per week. Hence, it contains five components: sugar, fat, whole meal, fruits & vegetables, and fish. Each component has a minimum score of 0 and a maximum score of 10. To obtain the total HDAS, scores for all five food groups were summed up to a maximum score of 50, where the highest score indicates the highest possible adherence to the dietary guidelines.

Sugar component: To calculate the intake of sugar we used the sugar propensity score developed earlier in IDEFICS^[[1]](#footnote-1)^. The intake frequency of all food items containing sugar (sweetened breakfast cereals, sweetened drinks, fruit juices, sweetened milk, sweet yoghurt and fermented milk beverages, fruits with added sugar, jam and honey, chocolate or nut based chocolate, candy bars, loose candies, marshmallows, biscuits, cakes, pastries, puddings, ice cream, milk or fruit based bars) have been summed up and divided by the total intake frequency of all food items reported. At the time of the development of the HDAS, the World Health Organization (WHO) stated that the intake should be less than 10E% from refined sugars. It was decided to set the highest score of 10 if the intake frequency of sugar containing food items was 10% of the total intake frequency or less. The quartiles of the sugar propensity score were counted. The upper limit of the lowest quartile (15.7%) was used to establish the score of “0”. The scores between 10% and 15.7% were equally distributed in steps by 0.6%. Children with a sugar propensity score above the first quartile (15.7%) received the score of zero (Table A1).

| **Table A1.** Scoring of the sugar component | |
| --- | --- |
| Proportion of sugar containing food items | Score |
| <= 10% | 10 |
| >= 10% to <10.6% | 9 |
| >= 10.6% to <11.3% | 8 |
| >= 11.3% to <11.9% | 7 |
| >= 11.9% to <12.6% | 6 |
| >= 12.6% to <13.2% | 5 |
| >= 13.2% to <13.8% | 4 |
| >= 13.8% to <14.5% | 3 |
| >= 14.5% to <15.1% | 2 |
| >= 15.1% to <15.7% | 1 |
| >= 15.7% | 0 |

Fat component: The principle is that healthier low fat versions of food items are set in relation to less healthier versions. The intake frequencies of food items generally low in fat (cooked vegetables, eggs, fish, meat and low fat dairy products and spread) was summed up. This sum was set in relation to the total intake frequency of both cooked and fried vegetables, eggs, fish, meat and low and high fat dairy products and spread. The proportion of the total intake frequency of low fat food items/week of the total intake frequency of food items containing fat per week was calculated according to the formula ‘total intake frequency of healthy food items a week / total intake frequency of all food items a week x 100’. For 100% low fat food items the score 10 was given and for less than 10% the score 0 was given. The scores between zero and 10 were distributed over equidistant categories of the healthy food item score (Table A2).

Whole meal component: The proportion of the total intake frequency of “whole meal bread, dark roll, dark crispbread” of the total intake frequency of bread (“white bread, white roll, white crispbread” + “whole meal bread, dark roll, dark crispbread”) was counted. The scoring was done in the same way as for fat, i.e. for 100% whole meal food items the score 10 was given and 0 for less than 10%. Similar to the fat score, the whole meal scores between zero and 10 were distributed over equidistant categories of the healthy food item score (Table A2).

| **Table A2.** Scoring of the fat and whole meal components | |
| --- | --- |
| Proportion of healthy food items | Score |
| = 100% | 10 |
| >= 90% to < 100% | 9 |
| >= 80% to < 90% | 8 |
| >= 70% to < 80% | 7 |
| >= 60% to < 70% | 6 |
| >= 50% to < 60% | 5 |
| >= 40% to < 50% | 4 |
| >= 30% to < 40% | 3 |
| >= 20% to < 30% | 2 |
| >= 10% to < 20% | 1 |
| < 10% | 0 |

Fruits and vegetables component: The total fruit and vegetable intake frequency was calculated as the intake frequency of fresh fruits without added sugar + half of the intake frequency of fresh fruits with added sugar + cooked vegetables, potatoes and beans + legumes + raw vegetables. Because parents had no control over meals eaten at pre-schools and schools estimations were made for these missing meals. According to the question “How often does your child usually eat at pre-school or school meals?” the amount of meals eaten at pre-school or school was calculated. The assumption was that children eat breakfast, lunch, dinner and at least one snack during the day, which adds up to 28 meals a week. The intake frequency per week reported in the FFQ was set in relation to the missing meals eaten in pre-school or school and converted into the amount the child would have eaten if school meals were included. The rationale behind the scoring for fruit and vegetables was that the recommendation for fruits and vegetables are five portions a day, i.e. 35 portions a week. The number of 35 portions or more was considered as optimal and received the highest score of 10. Less than two portions per week was scored as zero. The scores between zero and 10 were distributed over equidistant categories of the healthy food item score (Table A3).

| **Table A3.** Scoring of the fruits & vegetables component | |
| --- | --- |
| Intake frequency of fruits & vegetables per week | Score |
| >= 35 | 10 |
| >= 31.4 to < 35 | 9 |
| >= 27.6 to < 31.4 | 8 |
| >= 24 to < 27.6 | 7 |
| >= 20.4 to < 24 | 6 |
| >= 16.8 to < 20.4 | 5 |
| >= 13 to < 16.8 | 4 |
| >= 9.4 to < 13 | 3 |
| >= 5.8 to < 9.4 | 2 |
| >= 2.0 to < 5.8 | 1 |
| < 2.0 | 0 |

Fish component: The total intake frequency for fish was calculated in a similar way as for fruits and vegetables. The parents reported the intake of cooked and fried fish which was added and extrapolated for the missing meals. Fish intake was scored as the highest at an intake of 2.5 times/week; a value which corresponds to the recommendation of 2-3 portions a week. Zero fish intake was scored as zero. The remaining interval between 0 and 2.5 times/week was subdivided into nine intervals of size 0.3 times/week (Table A4).

| **Table A4.** Scoring of the fish component | |
| --- | --- |
| Intake frequency of fish/week | Score |
| >= 2.5 | 10 |
| >= 2.25 to < 2.5 | 9 |
| >= 2 to < 2.25 | 8 |
| >= 1.75 to < 2 | 7 |
| >= 1.5 to < 1.75 | 6 |
| >= 1.25 to < 1.5 | 5 |
| >= 1 to < 1.25 | 4 |
| >= 0.75 to < 1 | 3 |
| >= 0.5 to < 0.75 | 2 |
| >= 0.25 to < 0.5 | 1 |
| < 0.25 | 0 |

1. Lanfer A, Knof K, Barba G, Veidebaum T, Papoutsou S, de Henauw S, Soos T, Moreno LA, Ahrens W, Lissner L: **Taste preferences in association with dietary habits and weight status in European children: results from the IDEFICS study**. *International journal of obesity* 2012, **36**(1):27-34. [↑](#footnote-ref-1)
